# RNA Sequencing in Comparison to Immunohistochemistry for Measuring Cancer Biomarkers in Breast Cancer and Lung Cancer Specimens

**DOI:** 10.3390/biomedicines8050114

**Published:** 2020-05-09

**Authors:** Maxim Sorokin, Kirill Ignatev, Elena Poddubskaya, Uliana Vladimirova, Nurshat Gaifullin, Dmitriy Lantsov, Andrew Garazha, Daria Allina, Maria Suntsova, Victoria Barbara, Anton Buzdin

**Affiliations:** 1 Institute of Personalized Medicine, I.M. Sechenov First Moscow State Medical University, 119048 Moscow, Russia; sorokin@oncobox.com (M.S.); podd-elena@ya.ru (E.P.); allina_dasha@mail.ru (D.A.); suntsova86@mail.ru (M.S.); 2 Omicsway Corp., Walnut, CA 91789, USA; garazha@oncobox.com; 3 Shemyakin-Ovchinnikov Institute of Bioorganic Chemistry, 117997 Moscow, Russia; uliana.bagina@gmail.com; 4 Karelia Republic Oncological Hospital, 185000 Petrozavodsk, Russia; kirillignatev@bk.ru; 5 Vitamed Oncological Clinical Center, 121309 Moscow, Russia; 6 Faculty of Fundamental Medicine, Lomonosov Moscow State University, 119991 Moscow, Russia; gaifulin@rambler.ru; 7 Kaluga Regional Oncological Hospital, 248007 Kaluga, Russia; lantsov@mail.ru; 8 Oncological Dispensary of the Republic of Karelia, 185002 Petrozavodsk, Russia; evika9@rambler.ru; 9 Moscow Institute of Physics and Technology, 141701 Moscow, Russia

**Keywords:** transcriptomics, RNA sequencing, immunohistochemistry, molecular diagnostics, biomarkers detection, clinical oncology, targeted therapies, personalized medicine, lung cancer, breast cancer, trastuzumab, NCT03521245, bioinformatics

## Abstract

RNA sequencing is considered the gold standard for high-throughput profiling of gene expression at the transcriptional level. Its increasing importance in cancer research and molecular diagnostics is reflected in the growing number of its mentions in scientific literature and clinical trial reports. However, the use of different reagents and protocols for RNA sequencing often produces incompatible results. Recently, we published the Oncobox Atlas of RNA sequencing profiles for normal human tissues obtained from healthy donors killed in road accidents. This is a database of molecular profiles obtained using uniform protocol and reagents settings that can be broadly used in biomedicine for data normalization in pathology, including cancer. Here, we publish new original 39 breast cancer (BC) and 19 lung cancer (LC) RNA sequencing profiles obtained for formalin-fixed paraffin-embedded (FFPE) tissue samples, fully compatible with the Oncobox Atlas. We performed the first correlation study of RNA sequencing and immunohistochemistry-measured expression profiles for the clinically actionable biomarker genes in FFPE cancer tissue samples. We demonstrated high (Spearman’s rho 0.65–0.798) and statistically significant (*p* < 0.00004) correlations between the RNA sequencing (Oncobox protocol) and immunohistochemical measurements for *HER2/ERBB2*, *ER/ESR1* and *PGR* genes in BC, and for *PDL1* gene in LC; AUC: 0.963 for HER2, 0.921 for ESR1, 0.912 for PGR, and 0.922 for PDL1. To our knowledge, this is the first validation that total RNA sequencing of archived FFPE materials provides a reliable estimation of marker protein levels. These results show that in the future, RNA sequencing can complement immunohistochemistry for reliable measurements of the expression biomarkers in FFPE cancer samples.

## 1. Introduction

Both mRNA and protein levels can be used for interrogating gene expression in cancer tissues, both types of analysis having their advantages and limitations [1]. The protein level more closely reflects the cancer phenotype because these are proteins that execute major intracellular molecular functions. However, the mRNA and protein levels for known genes strongly correlate in the mammalian cells, so that mRNA levels explain ~84% of the variance in protein expression [2]. This has been also confirmed in different organisms by strong correlations between the mRNA and ribosomal footprinting or quantitative proteomics data (r range 0.59–0.89) [3,4,5]. 

Accurate, inexpensive, and reproducible high-throughput methods of quantitative proteomics are still under development [6]. However, there are many practical ways of measuring the expression of single proteins in tumor tissues, like immunohistochemistry, which has become a commonly used technique in clinical laboratory diagnostics [7]. Cancer transcriptomics provide direct analysis of RNA concentrations in tumor biosamples [8]. Transcriptomics have an advantage of being an approach unparalleled in terms of generation of high-throughput gene expression data due to the use of robust and relatively non-expensive experimental protocols applicable for the analysis of minute amounts of fresh or fixed cancer biomaterials [9]. The analysis of expression levels per single gene using such an approach is becoming a relatively cheap and easy task. 

RNA sequencing is considered a gold standard approach in modern transcriptomics [10,11]. Various RNA sequencing platforms have been used for gene expression profiling in human cancers, including Illumina [12], Ion Torrent/Proton [12], and Oxford Nanopore [13]. They utilize different equipment and physical principles for detecting output signals, but also various library preparation protocols, including different enzymes and numbers of PCR cycles [14]. This diversity results in dramatic batch effects and incompatibility of the outputs obtained using different platforms, reagents, and kits [15,16], which is why experimental gene expression profiles are generally compared among those obtained using the same platform [16]. In most cancer biology applications, gene expression in the tumor is compared with the normal samples. Case-to-normal gene expression ratios can be evaluated per se [17,18]. Alternatively, pools of differentially regulated genes can be analyzed systemically and systematically, e.g., by assessing enrichments of Gene Ontology (GO) terms [19,20] or interrogating activation of molecular pathways [21,22,23]. 

However, effort should be made to compare only compatible data. Several collections of RNA sequencing profiles have been published for normal human tissues. Ideally, they should represent tissues from healthy donors screened in a single assay with the same equipment and reagents. The biggest published dataset GTEx [24] (11,688 samples), however, lacks publicly available data on the donors’ exact age and requires complicated registration steps that cannot be performed by many researchers. There are also some basal contamination issues reported recently for GTEx [25]. Other relevant databases are freely accessible and include age information: The Cancer Genome Atlas, TCGA [26] (625 samples), ENCODE [27] polyA RNA-seq (41 samples), and ENCODE total RNA-seq (92 samples). Unfortunately, they also lack some of the previously mentioned features. In TCGA, the norms are adjacent to surgically removed tumors [28], but they can be not physiologically normal because of multiple pathologic effects tumors exert on the neighboring tissues, like inflammation [29], altered vascularization [30], and growth factors/cytokine balances [31]. In ENCODE, datasets were generated for the autopsy normal tissues using different library preparation methods, but they only include 1–4 samples per tissue type (including both male and female donors) and cannot form statistically significant reference groups in most of the cases. Finally, we recently published another atlas of normal tissue expression profiles termed the Oncobox Atlas of Normal Tissue Expression (ANTE) [32]. It has statistically significant reference groups for 20 human tissues/organs, and represents 142 solid tissue samples from human healthy donors killed in road accidents and 17 blood samples from healthy volunteers. The expressions were profiled in the experiments using the same reagents and protocols. 

However, very different RNA sequencing results can be obtained, depending on the source of clinical biomaterials. For fresh tissue specimens, high-integrity RNAs may be isolated, resulting in longer sequencing reads. For formalin-fixed paraffin embedded (FFPE) tissue samples, significantly degraded RNA preparations can be obtained, typically resulting in 25–50 bp single end reads [32]. While the read length depends on sequencing strategy and short reads could theoretically be obtained also from fresh-frozen tissues, storage in FFPE can potentially alter gene body coverage. This may lead to lower coverage for either 3’ or 5’ end of a gene [33]. Still, previous studies comparing FFPE vs. fresh-frozen samples obtained from the same tissues demonstrated lower (yet, comparable) gene body coverage for both storage techniques [34]. 

RNA reads are mapped to genes, while excluding ambiguous mapping entries, and the relative gene expression is then calculated. Working with degraded RNAs is problematic for the analysis of fused oncogenes because of too short reads that cannot be confidently mapped to fusion sites [35]. This is also the case for the analysis of differential alternative splice sites, because FFPE RNAseq results in lower percent of split-mapped reads when compared to RNAseq of fresh-frozen tissues [34]. However, degraded RNAs from FFPE specimens can provide high-quality expression profiles that cluster together with the samples from high-integrity RNAs of the same tissue, as shown by the ANTE project [32]. 

Here, we publish new original clinically and immunohistochemistry-annotated 39 breast cancer (BC) and 19 lung cancer (LC) RNA sequencing profiles, fully compatible with the Oncobox Atlas of Normal Tissues (ANTE). We performed the first correlation study of RNA sequencing and immunohistochemistry-measured expression profiles for the clinically actionable biomarker genes in FFPE cancer tissue samples. For *HER2/ERBB2*, *ER/ESR1*, and *PGR* genes in BC and for *PDL1* gene in LC, we demonstrated high and statistically significant correlations between the RNA sequencing (Oncobox protocol) and immunohistochemical measurements. 

These results show that RNA sequencing, at least if the Oncobox Atlas protocol for library preparation, data mapping, and normalization is followed, in the future, can complement immunohistochemistry for reliable measurements of the expression cancer biomarkers in FFPE samples. In addition to the FFPE data, we also observed a good correlation between RNA sequencing data and immunohistochemistry for the freshly frozen BC samples from the TCGA project database [36] with known HER2, ER, and PGR statuses.

## 2. Materials and Methods

### 2.1. BC Biosamples

All experimental biosamples of tumor tissues were formalin-fixed and embedded into paraffin blocks (FFPE). All biosamples were evaluated by a pathologist to confirm the tumor tissue origin and only the specimens with the content of tumor cells greater than 50% were investigated further. Of them, 16 breast cancer (BC) tissue samples were obtained from the Karelia Republic Oncological Hospital, Petrozavodsk, Russia, and 23 samples from Vitamed Oncological Clinical Center, Moscow, Russia. There were 30 primary tumors, 3 lymph node metastases, 2 scar metastases, 2 liver metastases, 1 brain metastasis, and 1 ovary metastasis. All the BC patients were women and the mean age was 51.9 years old (range 27–78 y.o.). Clinical annotation of the BC biosamples investigated is summarized in Table 1.

### 2.2. LC Biosamples

Nineteen lung cancer (LC) samples were obtained from the Vitamed Oncological Clinical Center, Moscow, Russia (*n* = 6) and from Kaluga Regional Oncological Hospital, Kaluga, Russia (*n* = 13). There were nine lung adenocarcinomas, seven squamous cell carcinomas, one adeno-squamous cell carcinoma, one small cell carcinoma, and one was unidentified. The patients were 17 men and 2 women, aged from 57 to 79 with the mean age of 67 years.

We collected information about the patients’ sex, age, diagnosis, and clinical history. Informed written consents to participate in the study and to include the results in this report were obtained from all patients. The consent procedure and the design of the study were approved by the ethical committees of both the Karelia Republic Oncological Hospital, Petrozavodsk, Russia and the Vitamed Oncological Clinical Center, Moscow, Russia. Clinical annotation of the LC biosamples investigated is summarized in Table 2. 

### 2.3. Preparation of Libraries and RNA Sequencing

To isolate RNA, 10 µM-thick paraffin slices were trimmed from each FFPE tissue block using a microtome. Eight paraffin slices were used for RNA extraction. RNA was extracted from FFPE slices using a QIAGEN RNeasy FFPE Kit following the manufacturer’s protocol. RNA 6000 Nano or Qubit RNA Assay kits were used to measure RNA concentration. RNA Integrity Number (RIN) was measured using Agilent 2100 bio-analyzer. For depletion of ribosomal RNA and library construction, KAPA RNA Hyper with an rRNA erase kit (HMR only) was used. Different adaptors were used for multiplexing samples in one sequencing run. Library concentrations and quality were measured using the Qubit dsDNA HS Assay kit (Life Technologies) and Agilent Tapestation (Agilent). Single-end RNA sequencing, 50 bp read length, for ~30 million raw reads per sample, was performed at Omicslab LLC, Moscow and at the Department of Pathology and Laboratory Medicine, University of California Los Angeles, using the Illumina HiSeq 3000 System. A data quality check was performed using the Illumina Sequencing Analysis Viewer and de-multiplexing was performed with Illumina Bcl2fastq2 v 2.17 software. Sequencing data were deposited to NCBI Sequencing Read Archive (SRA) under accession ID PRJNA565016 and PRJNA578290.

### 2.4. Processing of RNA Sequencing Data

RNA sequencing FASTQ files were processed with STAR aligner [37] in “GeneCounts” mode with the Ensembl human transcriptome annotation (build version GRCh38 and transcript annotation GRCh38.89). The STAR output contained expression levels for 58,233 individual genes. Ensembl gene IDs were converted to Human Gene Nomenclature Committee (HGNC, https://www.genenames.org/, database version from 13 July 2017) gene symbols. In total, expression level was calculated for 36,596 genes with the corresponding HGNC identifiers. 

### 2.5. Data Clustering

Log-transformed DESeq2 [38] normalized counts were used for hierarchical clustering analysis. The analysis was performed using R “ward.D” method. The dendrogram was visualized using a custom R script.

### 2.6. Immunohistochemistry

Immunohistochemistry assay for BC samples for HER2, ESR1, and PGR proteins was performed at the Clinical Diagnostic Laboratory of the Oncology Center of the Republic of Karelia, Russia, using antibody kits (Roche Diagnostics, USA) to identify the respective statuses of the tumors. For HER2, the output statuses were: (i) baseline staining (0), (ii) “+” (1), (iii) “++” (2), and (iv) “+++” (3). The “++” and “+++” statuses were confirmed using ISH DNA Probe Cocktail assay (Roche Diagnostics, Indianapolis, IN, USA). For ESR1 and PGR status, 0–8 grades were used.

LC biosamples were profiled at Unim Laboratory, Moscow (http://new.unim.su) for PDL1 protein expression. Hematoxylin-Eosin and PD-L1(ZR3) antibody (Sigma-Aldrich, USA) staining was used to assess the tumor statuses. The following output measures were used: (i) no cell membrane staining in biosample or staining of up to 1% of cells, (ii) staining of 1%–50% of cells, (iii) staining of 50%–100% of cells.

### 2.7. Literature Gene Expression Data

To compare freshly frozen tissue RNA sequencing and IHC data, we extracted all BC gene expression profiles with IHC-confirmed receptor status from The Cancer Genome Atlas project (TCGA), using the R “TCGAbiolinks” package [36]. In total, we analyzed 634 samples with confirmed HER2 status, 924 samples with confirmed ESR1 status, and 922 samples with confirmed PGR status. Identifiers of samples included in the analysis are given in Supplementary Table S1.

### 2.8. Statistical Analysis

Statistical analysis was performed using R software. Area under the receiver-operator curve was calculated using ROCR package. For the ROC-AUC analysis of the experimental data we used threshold >2 for separating ESR1-positive and PGR-positive cases from corresponding negative groups, according to [39]; HER2 “+++” were considered as HER2-positive, according to [40]; and tumors with more than 1% of cells stained with PD-L1 were considered as PD-L1-positive, according to [41]. Spearman’s correlation coefficient was used to test the significance of the correlation. Trendlines and 95% confidence intervals were built using stat_smooth function of ggplot2 package. The log rank test was used for survival analysis.

## 3. Results

### 3.1. RNA Sequencing Data

In this study, we investigated correlations between gene expression profiles established for formalin-fixed paraffin-embedded (FFPE) tissue biosamples, using RNA sequencing data and immunohistochemistry (IHC) staining. To this end, we experimentally profiled 39 breast cancer (BC) and 19 lung cancer (LC) FFPE tissue samples, using RNA sequencing; original data were deposited to NCBI sequencing read archive under accession number PRJNA565016.

We used the same protocol as for generating the Oncobox Atlas of RNA sequencing profiles of normal human tissues derived from healthy donors [32]. We found that application of the coverage threshold of 2.5 million mapped reads resulted in tissue specific clustering, whereas for the profiles with lower number of mapped reads, we observed biased clustering. In this study, we used the same sequencing and data processing and filtering protocol. All the current 39 breast cancer and 19 lung cancer RNA sequencing profiles passed the 2.5 million threshold (Table 1 and Table 2) and were analyzed further. The number of uniquely mapped reads appeared to be ranged from 3.96 to 20.54, which is common for sequencing of the RNA derived from FFPE [9,33].

**Figure 1:**
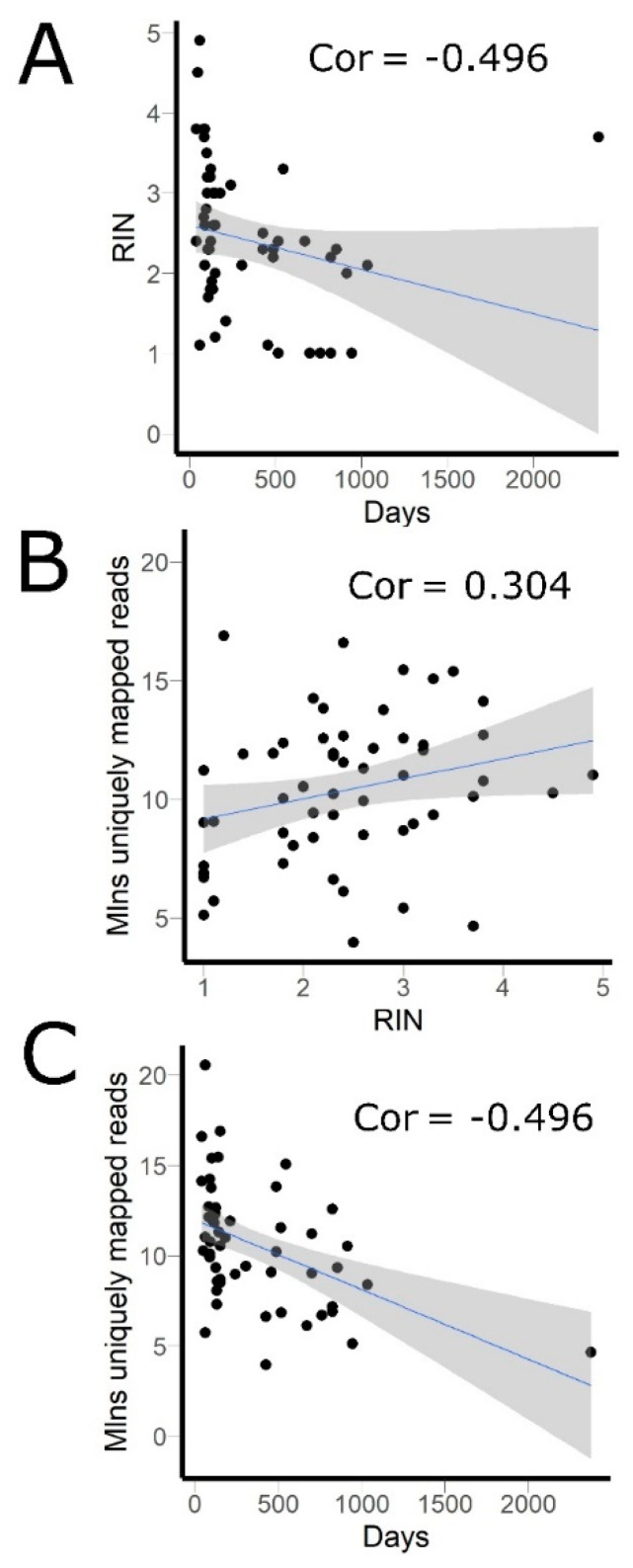
Effect of time interval between paraffinization and analysis (in days) on the quality of the sample. (**A**) RIN vs. time between paraffinization and analysis (Days): Spearman’s rho = −0.496 (*p*-value = 0.00012). (**B**) RIN vs. number of uniquely mapped reads per sample: Spearman’s rho = 0.304 (*p*-value = 0.022). (**C**) Time between paraffinization and analysis (Days) vs. number of uniquely mapped reads per sample: Spearman’s rho = −0.496 (*p*-value = 0.0001). Grey zone indicates 95% confidence interval for the trendlines. RIN—RNA integrity number, mln—million, cor—correlation coefficient.

**Figure 2:**
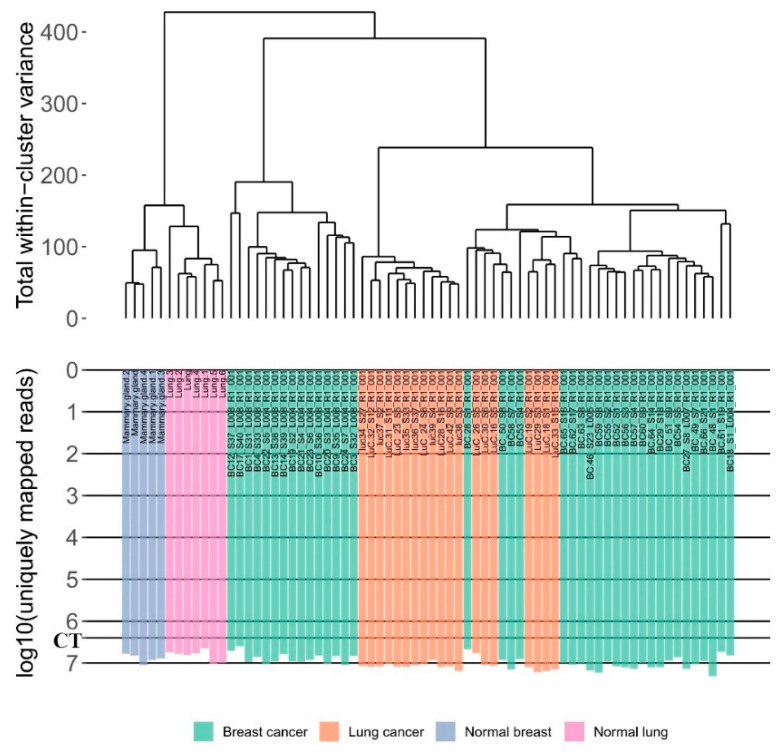
The hierarchical clustering dendrogram of experimental RNA sequencing profiles of breast and lung cancer and corresponding normal tissues from the ANTE database. Gene expression data were used to calculate Euclidean distances between the samples. The color markers indicate tissue types. The lower scale indicates the number of uniquely mapped reads. ‘CT’ denotes the coverage threshold of 2.5 million uniquely mapped reads.

The samples investigated were stored as FFPE tissue blocks in the Clinical Diagnostic Laboratory for 1–79 months before extraction of RNA (Figure 1). They had RNA integrity number (RIN) values ranging from 1 to 4.9, where lower RIN generally corresponded to more degraded RNA (Figure 1). We found significant correlation between the time from paraffinization to RNA extraction in days and the values of RIN (Spearman’s rho = −0.496 (*p*-value = 0.00012); Figure 1A). However, low RIN and samples’ age turned out not to be an informative marker of the insufficient number of gene-mapped reads, and all samples with 1 ≤  RIN  ≤  2 passed the coverage threshold as well (Figure 1B,C). All tumor gene expression profiles investigated were clustering mostly on a tissue-specific basis, thus confirming that they are of quality sufficient for analysis (Figure 2).

**Figure 3:**
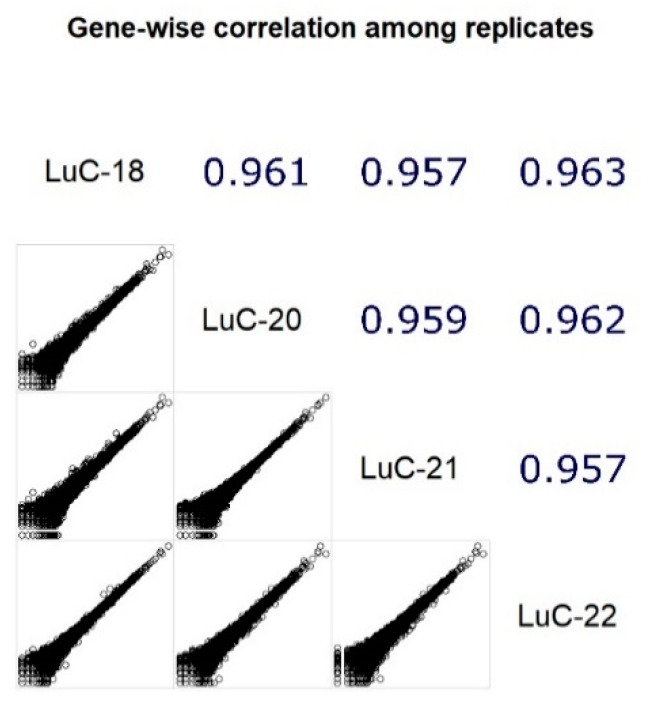
Correlation plot for four technical replicates (different slices from the same FFPE block) obtained from lung cancer tissue specimen. The samples were sequenced and processed separately. Top diagonal shows correlation coefficients (Spearman’s rho). Bottom diagonal shows pairwise plots for gene expression values.

We then assessed reproducibility of gene expression profiles by performing RNA sequencing for four different slices from the same FFPE tissue block (LC specimen LuC-18, see Table 2). The resulting four replicate samples were blinded and separately sent for sequencing. For all replicates, we observed high pairwise correlation coefficients (Spearman’s rho 0.96) between gene expression values (Figure 3). We, therefore, concluded that the RNA sequencing profiles obtained were reproducible for this sample. 

### 3.2. Comparison of RNA Sequencing and Immunohistochemistry Staining Results

**Figure 4:**
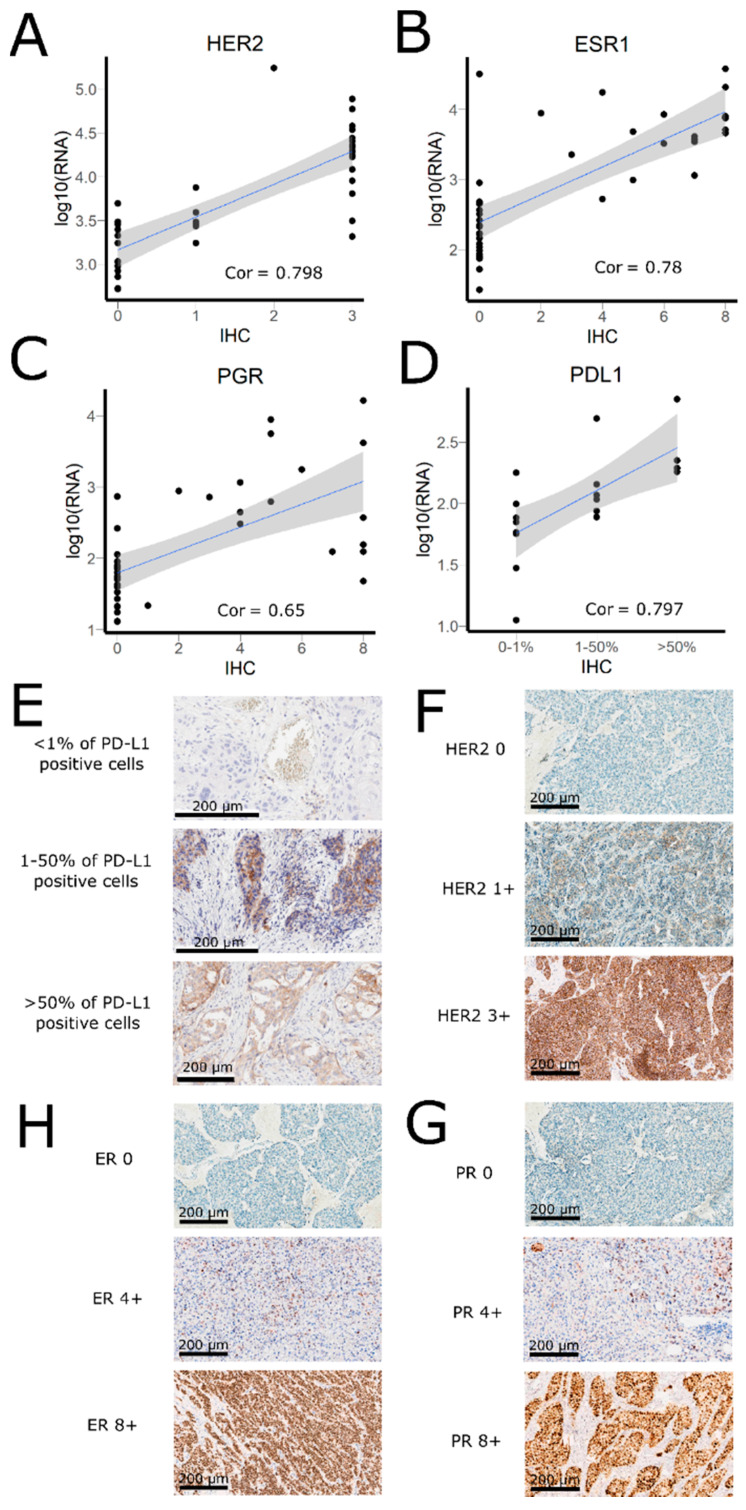
IHC results vs. mRNA level measured by NGS RNA sequencing: (**A**) HER2: correlation coefficient (Spearman’s rho) = 0.798 (*p*-value = 6.9 × 10^−10^); (**B**) ESR1: correlation coefficient (Spearman’s rho) = 0.777 (*p*-value = 3.8 × 10^−9^); (**C**) PGR: correlation coefficient (Spearman’s rho) = 0.653 (*p*-value = 4.9×10^−6^); (**D**) PD-L1: correlation coefficient (Spearman’s rho) = 0.797 (*p*-value =4.4 × 10^−5^). Grey zone indicates 95% confidence interval for the trendlines; (**E**) PD-L1 IHC staining examples. (**F**) HER2 IHC staining examples; (**H**) ER (ESR) IHC staining examples; (**H**) PR (PGR) IHC staining examples. Cor—correlation coefficient.

We then compared expressions of clinically actionable biomarker genes measured by RNA sequencing using Oncobox protocol (same as used previously to generate the Oncobox Atlas of Normal Tissues Expression [32]) and by immunohistochemistry (IHC). For the 39 BC specimens, HER2 (ERBB2), ER (ESR1), and PR (PGR) protein levels were measured by IHC. For 19 LC specimens, PDL1 protein levels were measured by IHC. Only clinically approved protocols and reagent sets were used for the IHC measurements. We then compared these results with the corresponding gene expression values obtained from RNA sequencing data. We found that the gene expression values were highly congruent with the IHC-measured protein levels for all four genes under investigation. The highest correlations were observed for *PDL1* expression in LC (Spearman’s rho = 0.797, *p* = 0.00004), *HER2* expression in BC (Spearman’s rho = 0.798, *p* = 6.9 × 10^−10^), and *ESR1* expression in BC (Spearman’s rho = 0.777, *p* = 3.8 × 10^−9^), while correlation with *PGR* in BC was lower yet still highly statistically significant (Spearman’s rho = 0.653, *p* = 4.9 × 10^−6^; Figure 4). 

**Figure 5:**
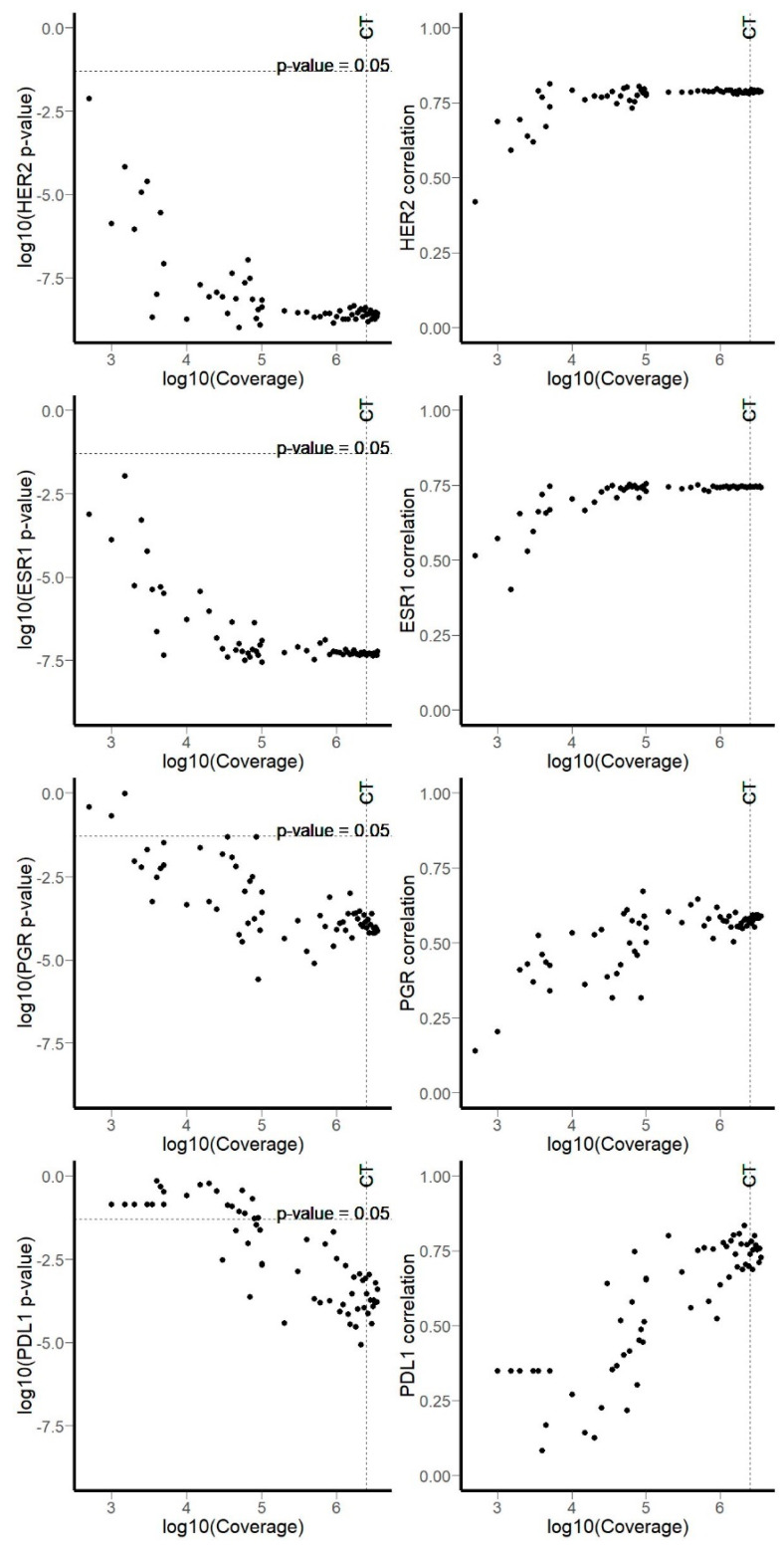
Computational simulation of gene-mapped reads coverage using random reads permutations. Left panels: *p*-value for Spearman’s rho vs. coverage. Right panels: Spearman’s rho vs. coverage. “CT” indicates coverage threshold of 2,500,000 reads.

In order to determine minimal numbers of uniquely mapped reads per sample required for statistically significant correlations between IHC and RNA sequencing data, we simulated samples with decreased coverage by randomly selecting reads from each sequencing experiment. Simulated coverage was in the range between 500 and 3,500,000 mapped reads. For each value of simulated coverage, we then calculated correlation coefficient and p-value for RNA sequencing-based gene expression vs. IHC status. We found that 3.5 million of uniquely mapped reads per sample was enough to obtain significant correlation for all biomarkers investigated, but the thresholds for minimal number of uniquely mapped reads varied for different biomarkers. Reducing the coverage to as low as to 100,000 mapped reads was enough for reliable estimation of *HER2* and *ESR1* levels in breast cancer tissues, while not less than a million mapped reads was required for *PGR* (Figure 5). We had 19 lung cancer samples, which can be the reason for greater variability observed for PDL1 correlations across simulations. However, all correlation coefficients were significant in cases with more than 2,500,000 total coverage by gene-mapped reads (Figure 5).

**Figure 6:**
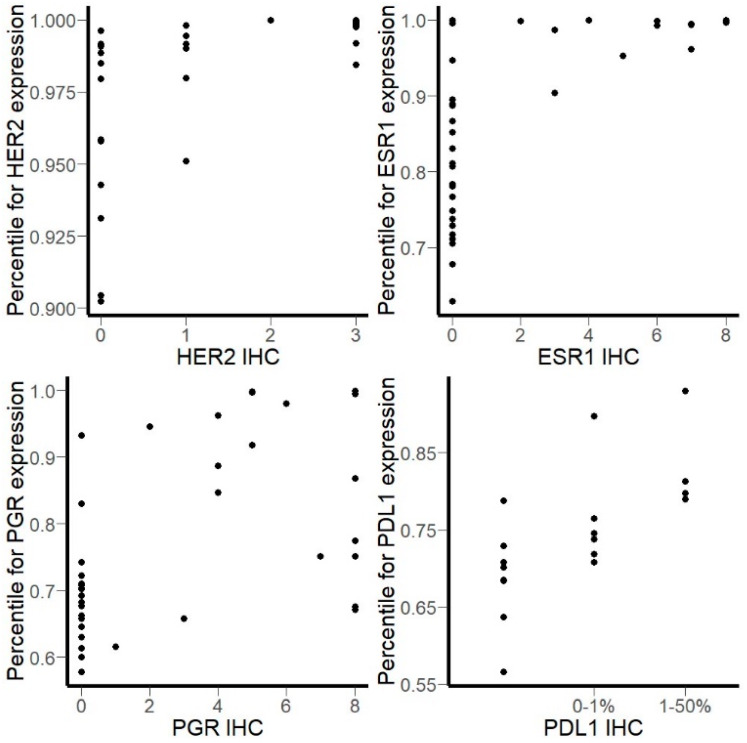
Percentile of gene counts for marker genes versus IHC (immunohistochemistry) score in breast and lung cancer samples.

To explain variability of minimal required coverage for different biomarkers, we calculated percentiles based on raw counts of each marker gene in every sample. We found that *HER2* was highly expressed at mRNA level even in IHC-negative breast cancer samples and was always in top 10% of most highly expressed genes (Figure 6). *ESR1* was in top 40% and *PGR* and *PDL1* in top 50% of the most strongly expressed genes. Therefore, higher mRNA abundance may be connected with the smaller coverage required for reliable estimation of gene expression, and vice versa.

### 3.3. Correlation of HER2, ER, and PGR Statuses Measured by RNA Sequencing and IHC for Freshly Frozen Tumor Samples

**Figure 7:**
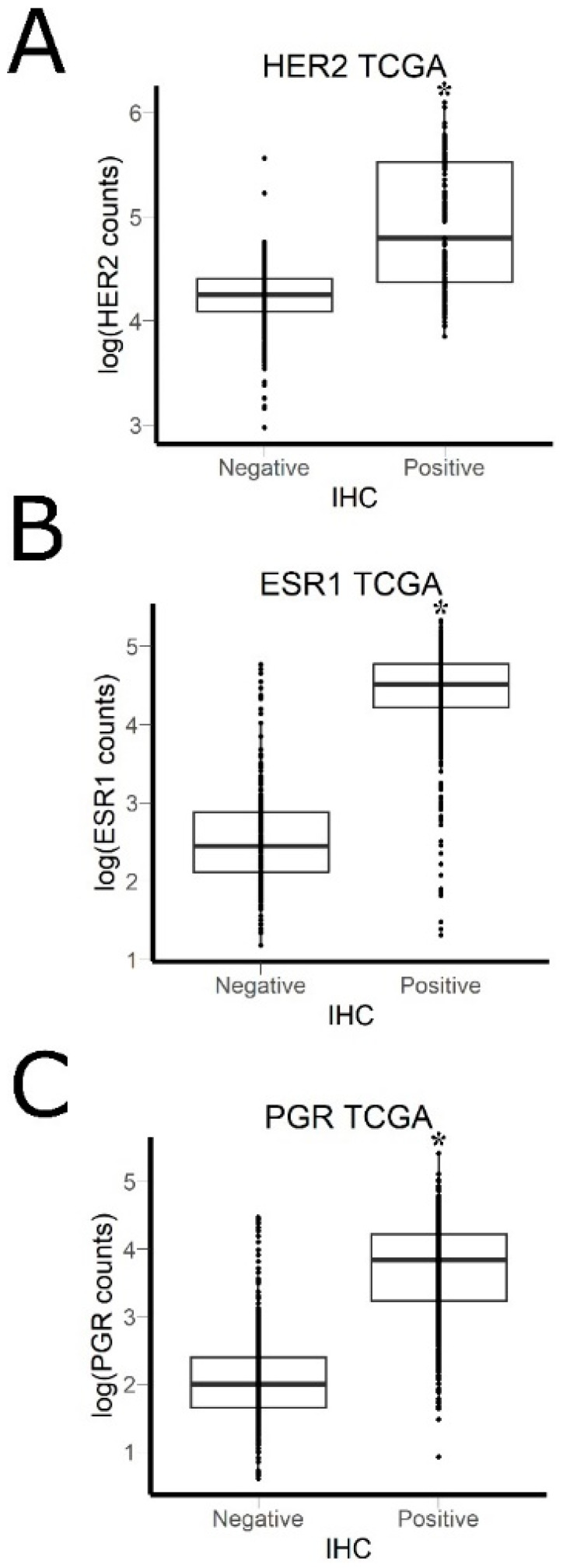
IHC results vs. mRNA level measured by NGS RNA sequencing in The Cancer Genome Atlas (TCGA) data: (**A**) HER2: area under the receiver-operator curve (AUC = 0.82); (**B**) ESR1: area under the receiver-operator curve (AUC = 0.96); (**C**) PGR: area under the receiver-operator curve (AUC = 0.92). * *p*-value < 2.2 × 10^−16^ (Wilcoxon rank-sum test).

To estimate the ability of RNA sequencing data from fresh-frozen tissue samples to predict IHC status, we extracted from The Cancer Genome Atlas project [36] all BC data with receptor status confirmed by IHC. We used binary classification (IHC negative/positive) for this analysis because only ~20% of TCGA BC samples were annotated with exact IHC scores for ESR1 and PGR. In total, we analyzed 634 samples with confirmed HER2 status, 924 samples with confirmed ESR1 status, and 922 samples with confirmed PGR status (Figure 7). We calculated area under the receiver-operator curve (AUC) value so that RNA sequencing data could be used to classify samples by the IHC status. AUC is the universal characteristic of biomarker robustness determined by its sensitivity and specificity [42]. This statistical approach is applicable to a wide range of different types of biomarkers in oncology [43,44,45,46,47,48]. AUC positively correlates with the quality of a biomarker and varies from 0.5 to 1. The standard discrimination threshold is 0.7 and the parameters with greater AUC are considered as high-quality biomarkers, and vice versa [49]. We obtained the following AUC values for TCGA data: 0.818 for HER2, 0.959 for ESR1, and 0.923 for PGR (Table 3). We then applied the same approach to our experimental FFPE data and obtained the following AUC: 0.963 for HER2, 0.921 for ESR1, 0.912 for PGR, and 0.922 for PDL1 (Table 3).

### 3.4. Correlation of HER2, ER, and PGR Expression Measured by RNA Sequencing versus Quantitative Proteomics

**Figure 8:**
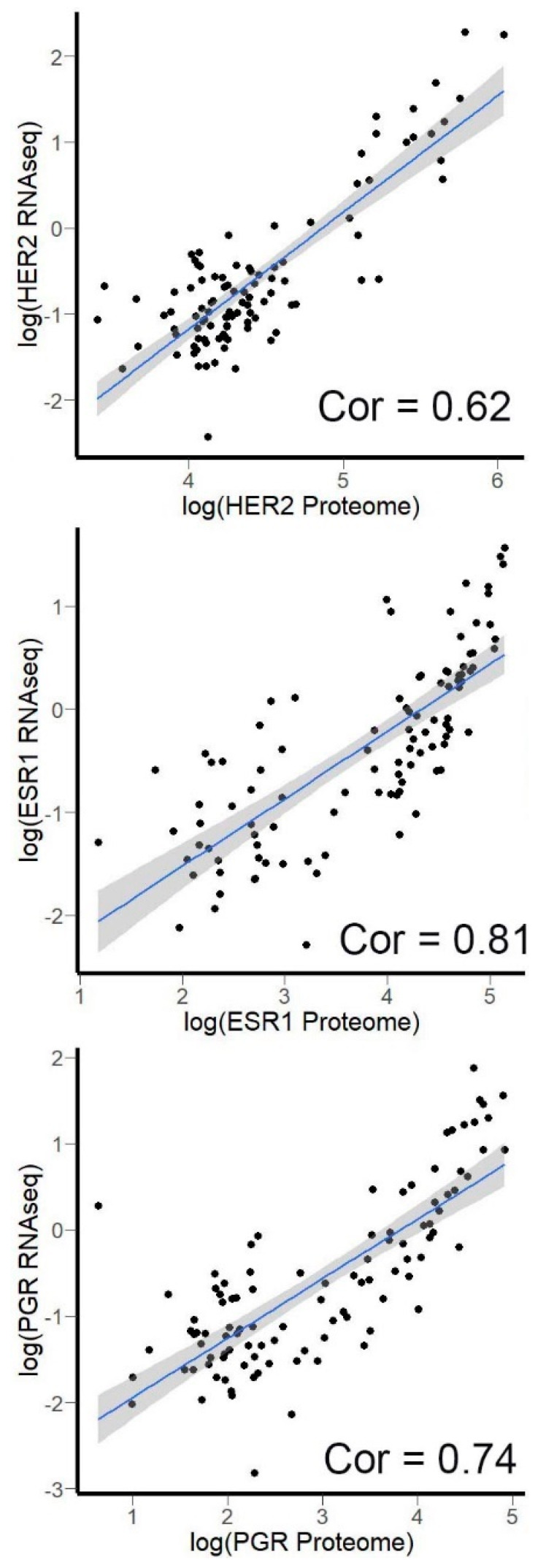
Proteomic results vs. mRNA level measured by NGS RNA sequencing in CPTAC data: (**A**) HER2: correlation coefficient (Spearman’s rho) = 0.62 (*p*-value < 2.2 × 10^−16^); (**B**) ESR1: correlation coefficient (Spearman’s rho) = 0.81 (*p*-value < 2.2 × 10^−16^); (**C**) PGR: correlation coefficient (Spearman’s rho) = 0.74 (*p*-value < 2.2 × 10^−16^); Grey zone indicates 95% confidence interval for the trendlines. Cor—correlation coefficient.

To investigate whether mRNA levels measured by RNA sequencing may serve as reliable markers of protein abundance, we analyzed quantitative proteomic profiles from The Clinical Proteomic Tumor Analysis Consortium (CPTAC) database [50,51]. The corresponding gene expression profiles for the same biosamples were extracted from the TCGA project database. For *HER2*, *ESR1*, and *PGR* analysis, we were able to identify 102 breast cancer samples with matched transcriptomic and proteomic profiles. Unfortunately, lung cancer samples were not annotated with expression of *PDL1* on protein level in CPTAC database. We, therefore, correlated mRNA and protein levels for the remaining biomarkers (Figure 8). The correlation coefficients for different biomarkers tested varied between 0.62 and 0.81.

## 4. Discussion

Immunohistochemistry (IHC) remains a method of choice for detecting expression of cancer biomarkers in most of clinical laboratories around the world [52,53,54]. However, RNA sequencing can be considered an even more accurate instrument for measuring the expression of biomarker genes, as this is the case for *PDL1* gene, whose expression positively correlates with patient’s response to anti-PD1/PDL1 immunotherapy [55]. It was previously shown that RNA sequencing of the same biosamples from FFPE materials and matched fresh-frozen tissues produce highly concordant expression profiles for breast [54,55] and ovarian cancers [56]. In addition, RNA sequencing generated coherent biological signals for the same FFPE samples when compared with targeted NanoString [57] or TaqMan PCR assays [58] for several biomarker gene products. That RNA sequencing can help accurately measure PDL1 has been reported previously for fourteen ovarian cancer tissue specimens [56], as well as the congruence of its concentration profiles obtained using IHC, qRT-PCR, and RNA sequencing, for both fresh-frozen and FFPE cancer tissue materials [56]. Another investigation of 437 samples from patients with non-small cell lung cancer revealed high correlation between PDL1 levels measured using IHC and qRT-PCR [57]. Recently, detection of expression of mRNA in cancer cells was thoroughly investigated using optical and electrochemical biosensors [58,59,60]; however, despite significant progress, these promising methods have not been introduced into wide laboratory practice yet.

Previous studies also investigated the possibility of using RNA sequencing data for predicting the IHC status of five conventional breast cancer biomarkers: ESR, HER2, PGR, Ki67, and Nottingham histologic grade (NHG) [61]. The authors observed good concordance between protein status determined by IHC and the level of corresponding gene expression determined by RNA sequencing. However, the main limitation of the study by Brueffer et al. is the use of fresh-frozen or RNA later preserved tissue [61]. Conroy et al. used FFPE samples for estimating PD-L1 level in various cancer types following targeted RNA sequencing approach, which was limited by the rather small number of genes analyzed in the experiment [55]. In our study, FFPE tissue blocks were investigated using total RNA sequencing. Such an approach allowed reliable estimation of cancer biomarkers and additionally provided gene expression data on a larger scale. 

Multiple layers of gene expression regulation, including post-transcriptional, translational, and post-translational, contribute to the proteomic landscape of the cell [62], and thus, may cause inconsistencies between results of RNA sequencing and immunohistochemistry measuring cancer biomarkers. However, ours and previous studies reported a high degree of concordance between these methods, at least for clinically relevant genes, thus providing evidence that RNA sequencing may complement IHC for measuring cancer biomarkers [55,61]. Although, it potentially may not be true for genes heavily regulated by post-transcriptional or post-translational modifications, and therefore, correlation between RNA sequencing and IHC should be independently validated for other biomarkers.

Here, we investigated correlations between the IHC- and RNA sequencing-measured expression profiles for four clinically actionable biomarker genes in 39 BC and 19 LC cancer biosamples. Among them, positive ESR1 and PGR status is crucial for the use of hormone therapy to treat BC, and HER2 status of 2 or 3 is an indication for targeted anti-Her2 therapeutic antibodies prescription in BC, e.g., trastuzumab [40]. In turn, PDL1 status is an important biomarker for immunotherapy in several cancer types, including lung cancer, where PDL1-positive staining of membranes of more than 50% of cancer cells serves as the key indication for prescription of PD1-specific immune check-point inhibitors, e.g., pembrolizumab, nivolumab, and atezolizumab [63]. We found that the results of RNA sequencing strongly correlate with the results obtained by IHC methods in different clinical laboratories. This suggests that, theoretically, RNA sequencing can be used to select the optimal treatment strategy for FFPE cancer tissue samples as an alternative or as an addition to IHC. In addition to simply profiling few clinical biomarker genes, RNA sequencing data enable identification of differentially expressed drug target genes [64] and measuring molecular pathway activation [21,23]. Among the others, this allows patient-oriented personalized ranking of cancer drugs with known molecular specificities [21,23,65].

However, different RNA sequencing platforms and protocols often generate incompatible results, and it is important for data reproducibility to define the experimental procedure and analytic pipeline used to obtain the results. This is especially important for comparisons with the expression in normal reference tissues [66]. To obtain and analyze RNA sequencing data, we followed strictly the procedure previously published for generating the atlas of human normal tissue transcriptomes (i.e., the Oncobox Atlas of Normal Tissue Expression) [32]. This made these two datasets fully compatible in terms of further data analysis. We also show that this experimental and analytic procedure ensures obtaining high-quality transcriptomic data that strongly correlate with the gene expression values measured by IHC. In addition to the FFPE data, we also observed a good correlation between the RNA sequencing data and the results of immunohistochemistry for the freshly frozen BC samples with known HER2, ER, and PGR statuses from the TCGA project database [36].

However, RNA sequencing provides expression levels for all genes, thus revealing much more information about a tumor, which could be applied synergistically in clinical practice. For example, high-throughput gene expression analyses were used during WINTHER [67] and Oncobox [68] clinical trials. Both trials used gene expression profiling of tumor biopsy for personalized prescription of targeted drugs to patients with advanced tumors. In addition, high-throughput gene expression profiling was previously used to select successful therapies for solid tumor patients, as described in several previous reports [69,70,71,72]. Moreover, nowadays, RNA sequencing can be performed for approximately 250 USD per sample, and this price tends to decrease further [73]. At the same time, single immunohistochemical staining procedure can cost up to 220 USD per sample [74]. Thus, with the rapid emergence of new biomarkers and their introduction into clinical practice, RNA sequencing can potentially become an at least equally useful and cost-effective solution.

## Figures and Tables

**Table 1:** Clinical and molecular annotation of the breast cancer biosamples.

Sample ID	Primary Tumor or Metastasis	Age	Stage	HER2 Score	ER Score	PR Score	Coverage (mln Mapped Reads)	RIN
BC-1	primary	39	T2N3aM0, IIIC	3	0	0	9.42	2.1
BC-10	primary	48	T2N0M0, II	3	0	0	6.70	1
BC-12	primary	60	T2N0M0, IIA	3	0	0	5.12	1
BC-13	primary	69	T2N3M0, III C	3	8	4	9.03	1
BC-14	primary	49	T2N2M0, IIIA	3	0	0	6.11	2.4
BC-17	primary	59	T4N2M0	3	7	2	3.96	2.5
BC-18	lymph node metastasis	47	T3N1M0, IIIA	3	0	0	6.62	2.3
BC-19	primary	48	T1N0M0, I	3	5	5	9.07	1.1
BC-20	lymph node metastasis	51	T2N0M0, II	3	0	0	10.22	2.3
BC-21	primary	49	T1N3M0, IIIC	3	0	0	9.34	2.3
BC-22	primary	47	T2N0M0, II	3	6	5	10.52	2
BC-23	primary	46	T2N2M0, IIIA	3	7	6	8.39	2.1
BC-24	primary	57	T2N0M0, IIA	3	6	4	11.21	1
BC-27	primary	44	T2N0M0	3	0	0	13.82	2.2
BC-28	ovary metastasis	53	T2N0M0, IIA	0	7	4	4.65	3.7
BC-29	primary	65	T4N3M1,IV	3	0	0	12.56	2.2
BC-3	primary	55	T2N1M0, IIIa	3	0	0	6.84	1
BC-4	primary	58	T2N1M0, IIB	3	0	0	7.17	1
BC-46	liver metastasis	27	T2N2M0	0	8	8	15.07	3.3
BC-48	relapse in the scar	36	T3N1M0	1	0	0	20.54	NA
BC-49	primary	54	T1N2M0	0	2	8	10.54	2
BC-50	primary	51	T2N0M0	0	0	0	8.49	2.6
BC-51	primary	38	T2N1M0	0	0	0	8.68	3
BC-52	primary	78	T1N2M0	1	4	8	11.92	1.7
BC-53	primary	50	T2N0M0	1	0	8	8.06	1.9
BC-54	primary	50	T2N0M0	0	0	0	7.30	1.8
BC-55	primary	71	T2N3M0	1	8	8	9.32	3.3
BC-56	primary	60	T1N1M1	0	0	8	12.66	2.4
BC-57	primary	55	T3N2M0	1	0	0	13.77	2.8
BC-58	lymph node metastasis	55	T1N0M0	0	7	7	14.24	2.1
BC-59	scar metastasis	61	T1N1M0	0	3	1	16.88	1.2
BC-60	primary	33	T2N1M0	2	0	0	10.03	1.8
BC-61	liver metastasis	38	T2N2M0	0	8	8	5.42	3
BC-62	brain metastasis	44	T2N0M0	0	0	0	10.99	3
BC-63	primary	66	T4N2M0	0	0	0	10.11	3.7
BC-64	primary	60	T3N3M0	1	0	0	12.71	3.8
BC-65	primary	42	T2N0M0	0	0	0	9.92	2.6
BC-66	primary	55	T3N1M0	3	3	3	8.96	3.1
BC-9	primary	57	T1N1M0, IIB	3	8	5	6.88	1

RIN—RNA integrity number, mln—million, NA—not assessed.

**Table 2:** Clinical and molecular annotation of the lung cancer biosamples.

ID	Histology	Age	Stage	Sex	Percent of PDL1 Positive Cells	Coverage (mln Mapped Reads)	RIN
LuC_16	squamous cell carcinoma	75	T3N2M1, IV	male	1%–50%	11.54	2.4
LuC_18	squamous cell carcinoma	63	T2N1M0	male	0	15.45	3
LuC_19	squamous cell carcinoma	65	T2N0M0	male	>50%	12.57	3
LuC_30	Unidentified	79	T2NXM0	male	>50%	11.01	4.9
LuC_31	adenocarcinoma	66	T3N2M0	male	1%–50%	10.27	4.5
LuC_32	adeno-squamous cell carcinoma	70	T2N1M0	male	>50%	12.14	2.7
LuC_33	squamous cell carcinoma	57	T3N0M0	male	0	14.12	3.8
LuC_42	adenocarcinoma	67	T1N1M0	male	1%–50%	11.9	1.4
LuC_23	adenocarcinoma	60	T2N0M0	male	0	12.06	3.2
LuC_24	adenocarcinoma	67	T2N0M0	male	0	10.77	3.8
LuC_26	small cell carcinoma	65	T3N2M0, IIIa	male	1%–50%	5.71	1.1
LuC_28	adenocarcinoma	76	T2N0M0	male	0	12.37	1.8
LuC_29	squamous cell carcinoma	65	T2N0M0	male	0	16.58	2.4
LuC_34	adenocarcinoma	62	pT1bN0M0	female	0	11.82	2.3
LuC_35	squamous cell carcinoma	75	T3N0M0	male	>50%	12.28	3.2
LuC_36	adenocarcinoma	57	pT2N0M0	male	1%–50%	11.3	2.6
LuC_37	squamous cell carcinoma	68	T3N1M0	male	0	11.93	2.3
LuC_38	adenocarcinoma	68	pT2aN2M0	male	1%–50%	15.38	3.5
LuC_39	adenocarcinoma	68	pT2pNXpM1	female	0	8.58	

RIN—RNA integrity number, mln—million, NA—not assessed.

**Table 3:** Area under the receiver-operator curve (AUC) for predicting IHC status using RNA sequencing data.

Protein	Experimental Dataset	The Cancer Genome Atlas
HER2	0.963	0.818
ESR1	0.921	0.959
PGR	0.912	0.923
PDL1	0.922	Not available

## Supplementary Materials

The following is available online at https://www.mdpi.com/2227-9059/8/5/114/s1, Table S1: TCGA BC data with IHC confirmed receptor status.
